# The Brazilian Native Fruits Araçá, Guabijú, and Guabiroba: A Brief and Integrative Review on Their Phenolic Composition and Analytical Methods

**DOI:** 10.3390/foods14162858

**Published:** 2025-08-18

**Authors:** Patrícia Gotardo Machado, Felipe Siqueira Molina, Milene Teixeira Barcia, Cristiano Augusto Ballus

**Affiliations:** Department of Food Science and Technology, Federal University of Santa Maria (UFSM), Santa Maria 97105-900, Brazil; patricia.machado@acad.ufsm.br (P.G.M.); felipe.molina@acad.ufsm.br (F.S.M.); milene.barcia@ufsm.br (M.T.B.)

**Keywords:** phenolic compounds, antioxidant capacity, mass spectrometry, liquid chromatography, biodiversity, Myrtaceae

## Abstract

Brazil has one of the greatest biodiversities in the world, with emphasis on the fruit family Myrtaceae, to which the native fruits guabijú (*Myrcianthes pungens* (O.Berg) D.Legrand), guabiroba (*Campomanesia xanthocarpa* (Mart.) O.Berg), and araçá (*Psidium cattleyanum* Sabine) belong. These fruits are promising sources of phenolic compounds, mainly phenolic acids, flavonoids, and tannins, with high antioxidant capacity and potential health benefits. This integrative review aimed to gather and analyze data on the phenolic composition of these fruits, as well as the analytical methods used for their extraction and characterization. The reviewed literature reveals that there is still a lack of in-depth studies, although some studies have already characterized the phenolic compounds in different parts of the fruits. The efficient extraction of phenolics, especially bound ones, requires techniques such as acid or alkaline hydrolysis. For their identification and quantification, the use of liquid chromatography (LC) coupled to mass spectrometry (MS) with mass analyzers such as triple quadrupole (QqQ) and quadrupole-time-of-flight (QToF), stands out. Knowledge of these fruits contributes to the development of functional ingredients and the conservation and appreciation of Brazilian biodiversity, thereby reinforcing the importance of expanding research on these fruits and exploring potential applications in the food, pharmaceutical, and cosmetic industries.

## 1. Introduction

Brazil stands out worldwide for its extraordinary plant diversity, with more than 45,000 known species, many of which are fruit-bearing and have significant potential for various applications [1,2]. This enormous biodiversity offers opportunities not only for the direct consumption of the fruits but also for the development of plant extracts used by the pharmaceutical, food, and chemical industries.

With its vast variety of fruits, the country ranks third in global fruit production, accounting for around 20% of the world’s total [3]. However, despite the abundance of native fruits, many of them remain underutilized, and their potential remains largely unknown [4]. The exploitation and valorization of these native fruits can play a crucial role in ensuring food and nutritional security, while also contributing to the conservation of biodiversity as well as the sustainable management of natural resources [5].

In the Brazilian state of Rio Grande do Sul, where these native fruits grow in adverse environmental conditions without the use of pesticides, they not only have significant economic potential but also make an essential contribution to biodiversity conservation [6].

**Figure 1:**
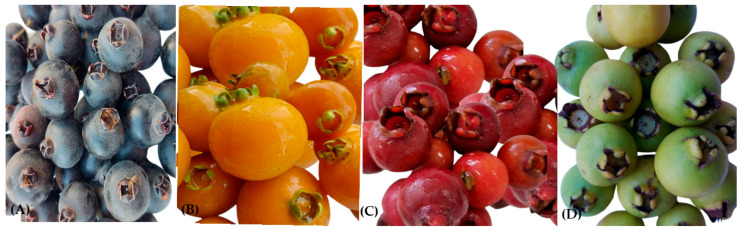
The native fruits of the Myrtaceae family presented in this review:guabijú, guabiroba, and araçás. (**A**) Guabijú (*Myrcianthes pungens* (O.Berg) D.Legrand), (**B**) Guabiroba (*Campomanesia xanthocarpa* (Mart. O.Berg), (**C**) Araçá red (*Psidium cattleyanum*), and (**D**) Araçá yellow (*Psidium cattleyanum* Sabine).

Among the native fruits, those of the Myrtaceae family are notable for their high nutritional value and the abundance of specialized metabolites, such as flavonoids, tannins, and phenolic acids, which possess antioxidant properties and offer several benefits to human health [7,8,9]. Among the many fruits belonging to this fruit family, guabijú (*Myrcianthes pungens* (O.Berg) D.Legrand), guabiroba (*Campomanesia xanthocarpa* (Mart.) O.Berg), and araçá (*Psidium cattleyanum* Sabine) stand out as new research interests in bioactive compounds (Figure 1).

Thus, this brief review aims to elucidate the phenolic compounds that have been characterized to date in the native fruits guabijú, guabiroba, and araçá, while also presenting the most efficient analytical techniques for their characterization.

## 2. Methodology

This study is an integrative review of a qualitative nature. Searches were conducted in Scopus, Science Direct, Taylor & Francis, and open-access databases of the National Library of Medicine, selecting scientific studies and books written in English and Portuguese from 2005 to 2025, totaling 20 years of publications available in the databases. The search was conducted between September 2024 and July 2025, using the terms: “*native fruits*”; “*antioxidant capacity*”; “*antioxidant activity*”; “*phenolic compounds*”; “*liquid chromatography*”; “*mass spectrometry*,” as well as the common names of the fruits “*guabijú*,” “*guabiroba*,” and “*araçá*,” and their scientific names: “*Myrcianthes pungens*”; “*Campomanesia xanthocarpa*”; “*Psidium cattleyanum*”. To further deepen the search, we used the terms individually and correlated. We selected the articles and books based on their direct relevance to native fruits of the Myrtaceae family, especially regarding their phenolic profile. We also considered review and experimental articles. These search and analysis criteria ensured that the selected studies met the objectives of this integrative review.

## 3. Fruits of the Myrtaceae Family

The Brazilian flora is characterized by great diversity, with native edible fruits produced by species of the Myrtaceae family having significant ecological importance [10]. Since most fruits of the Myrtaceae family develop under adverse environmental conditions, they are a source of specialized metabolites involved in plant defense, mainly biologically active polyphenols [11].

The composition of some genera of the Myrtaceae family includes phenolic compounds such as flavonoids, phenolic acids, tannins, stilbenes, coumarins, tocopherols, and carotenoids [12]. Studies also show that the fruits have antioxidant capacity and a significant content of anthocyanins [13]. Flavonoids, recognized as natural antioxidants, are the main bioactive compounds found in fruits of the Myrtaceae family [14,15].

Researchers have correlated the regular intake of fruits and vegetables with a reduced risk of several chronic diseases, such as heart disease, stroke, cancer, atherosclerosis, diabetes, Alzheimer’s disease, cataracts, and lung disorders [16,17]. Some native fruit species belonging to the Myrtaceae family exhibit anti-obesity activity and are associated with reduced complications [10].

According to Antonelo et al. [18], the fruits of the Myrtaceae family present antioxidant capacity in vitro by tests for estimation of antioxidant capacity DPPH (2,2-diphenyl-1-picrylhydrazyl), ABTS (2,2′-azino-bis (3-ethylbenzothiazoline-6-sulfonic acid)), and FRAP (Ferric Reducing Antioxidant Power). As in their research, Machado et al. [8] demonstrated that the peroxyl radical scavenging capacity (ORAC) increases the interest for their use in the food additive industry as antioxidants and natural dyes.

Many species of fruits from the Myrtaceae family are of great economic importance. They are recognized for their high nutritional value and for being sources of bioactive compounds, such as the araçá, the guabijú, and the guabiroba.

**Figure 2:**
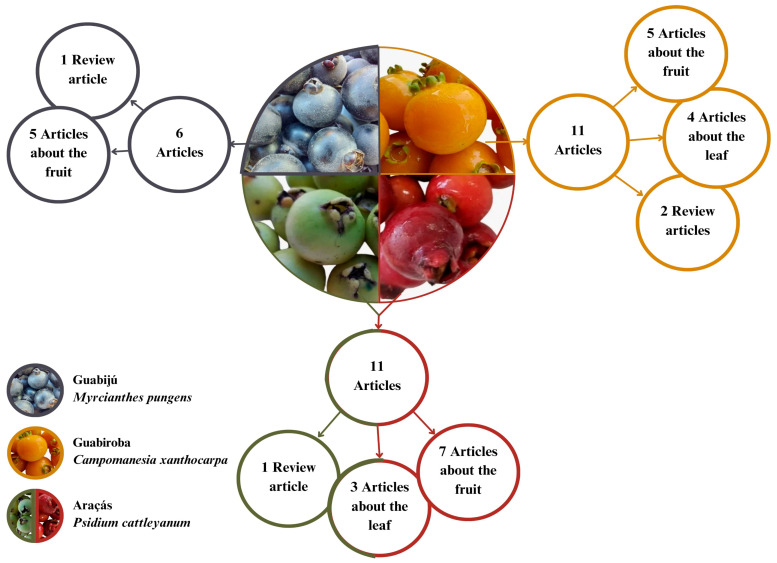
Studies available in the literature on the phenolic compounds present in araçá, guabijú, and guabiroba fruits.

Figure 2 presents a survey of data platforms on works already published on the fruits, which have some relationship with their phenolic composition and/or antioxidant capacity. It is noteworthy that the literature contains few studies on the subject.

## 4. Phenolic Compounds in Fruits and Their Analytics

Phenolic compounds are specialized metabolites produced primarily by plants; their molecules have one or more aromatic rings coupled to one or more hydroxyl groups, and they can be found in the form of simple molecules or polymerized compounds [19]. They range from simple compounds, such as phenolic acids, to complex compounds such as tannins, and their presence in plants makes them a vital ingredient in the human diet [20].

According to Alara et al. [21], phenolic compounds are found mainly in fruits, vegetables, greens, tea, wine, and coffee, and are responsible for the organoleptic characteristics of plant foods. Likewise, they are responsible for the bitterness of fruits due to their interaction with salivary glycoprotein, and may also be responsible for the color of many fruits and vegetables.

Phenolic compounds exist in different classes according to their structural characteristics, but they are also found in various forms depending on their association with the food matrix. These compounds can be present in soluble free forms, soluble esters, or conjugated and insoluble forms when linked to complex matrices [22].

**Figure 3:**
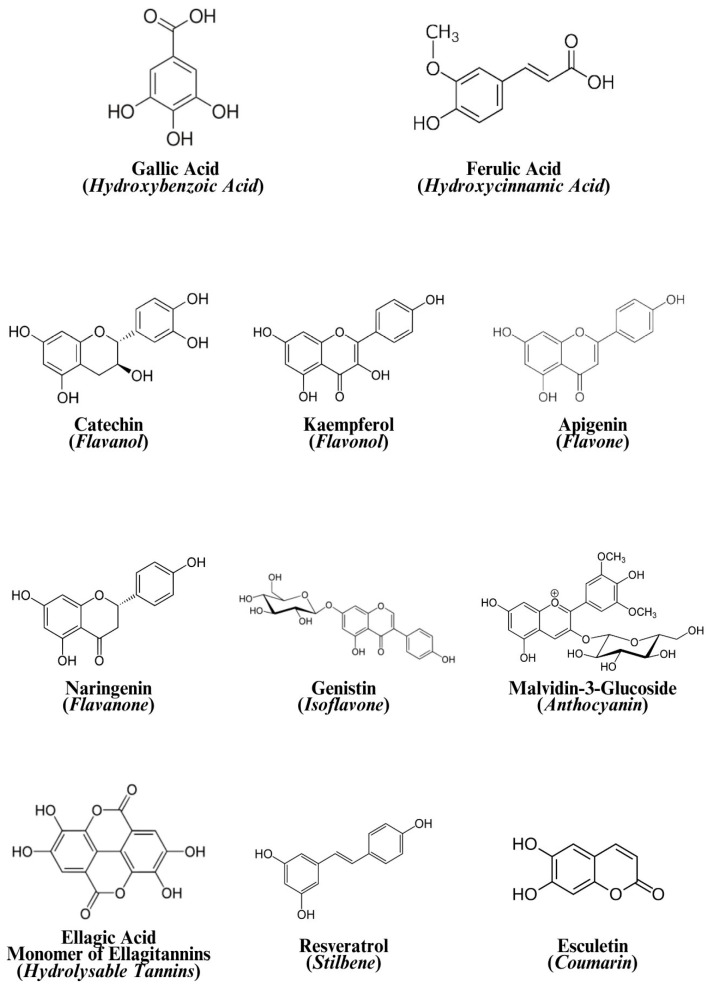
Structures of phenolic compounds representing each class.

Figure 3 presents an example of structures for each of the phenolic classes.

### 4.1. Extracting Bound Phenolic Compounds

In the past decade, most of the studies published regarding phenolics have covered the free soluble form. It is believed that this fact is due to the analytical complexity that some matrices impose during their extraction, as well as the difficulties in evaluating the results obtained.

To obtain plant extracts containing phenolic compounds, some studies indicate that it is necessary to use alkaline and acid treatments. According to Fazary et al. [23], the main variables of these chemical hydrolyses are the acid/base concentrations, hydrolysis time, and temperature. The acid treatment breaks the glycosidic bonds and solubilizes the sugars, but generally leaves the ester bonds intact. And according to Acosta-Estrada et al. [24], alkaline hydrolysis promotes the rupture of ester and ether bonds; both hydrolysis and the most common means of releasing bound phenolic compounds.

Soluble phenolics are usually extracted from the food matrix using different combinations of aqueous and organic solvents, as well as techniques optimized for specific soluble phenolic fractions. However, a large amount of bound insoluble phenolics may remain in the matrix after extractions by the methods mentioned above [25].

Conventional extraction methods are not sufficient to release phenolics bound to structural components. These compounds have demonstrated significantly higher antioxidant activity compared to soluble phenolics. Furthermore, they can survive the conditions of the human stomach and small intestine and reach the colon intact, where they are released and exert bioactivity [22,26].

Although bound phenolic compounds have already been reported in various foods, a common understanding and comparable protocols for their extraction, characterization, and evaluation are still lacking, which can be applied to different food matrices.

### 4.2. Characterization and Quantification of Phenolic Compounds

The characterization of phenolic compounds can be a complex task due to their diverse structures and the complex matrices in which they are contained. However, analytical chemistry offers techniques capable of separating these compounds and quantifying them in various detection systems, which have become essential for advancing research.

According to Solárová et al. [27], in the mid-20th century, bioactive compounds did not have an extensively recognized role; however, after the analytical advances in liquid and/or gas chromatography coupled with mass spectrometry, as well as other analytical techniques, it became possible to evaluate these phytochemical compounds and their biosynthetic pathways.

Liquid chromatography is a technique used to separate compounds present in a mixture. Mass spectrometry provides structural information about each compound analyzed. The combination of both technologies allows the qualitative and quantitative characterization of phenolic compounds.

High-performance liquid chromatography (HPLC) is the most versatile and widely employed type of elution chromatography, being used to separate and determine species in a variety of organic, inorganic, and biological materials [28]. In terms of scale, in an extreme, minute quantities smaller than a nanogram are separated and identified during analysis [29].

Mass spectrometry (MS) aims to identify a compound from the molecular or atomic masses of its constituents. Its characteristics have elevated it to a prominent position among analytical methods, with unparalleled sensitivity, detection limits, speed, and a diverse range of applications [30]. The information provided by mass alone may be sufficient for identifying elements and determining the molecular formula of an analyte, just as the fragmentation of ions can give information about their ionic structure [31]. Two mass/charge analyzers can be used to study phenolic compounds: the triple quadrupole (QqQ) and the quadrupole time-of-flight (QToF).

The triple quadrupole can be operated in sequential mode (MS/MS). When the equipment has this configuration, it indicates that, of the three quadrupoles, the second, represented by the lowercase letter, is the region where fragmentation occurs (collision cell). All ions are separated in the mass spectrometer according to their mass-to-charge ratio (*m*/*z*), where “*m*” represents the relative mass and “*z*” the number of charges, both treated as dimensionless quantities [30]. Still in sequential mode, it is possible to configure the scanning mode in which the equipment will operate. MRM, multiple reaction monitoring, operates as follows: the first and second quadrupoles are configured to select the masses of the analytes; therefore, scanning does not occur. The absence of scanning allows for a longer focus on precursor and fragment ions, thus increasing the sensitivity and selectivity of the method [30]. Due to its high sensitivity, it is frequently used in the quantification of phenolic compounds.

Time-of-flight (ToF) mass analyzers are frequently used in the identification of phenolic compounds due to their ability to provide comprehensive information on the exact molecular mass, molecular formula, and chemical structure of a given compound [32,33]. By obtaining this data, it is possible to identify the compounds that were analyzed. Using the generated fragments, the molecular mass, and the retention time of each compound, it is possible to search for compounds with the same characteristics, both manually in the literature and in databases using software, to identify an unknown compound.

## 5. Phenolic Compounds of Guabijú, Guabiroba, and Araçá

Native Brazilian fruits of the Myrtaceae family possess a vast diversity of phenolic compounds in their complex compositions. Despite belonging to the same fruit family, each fruit has its own characteristics, also distinguishing them from one another in their phenolic composition.

For a more detailed overview, Table 1 presents the studies that evaluated the phenolic compounds, in detail or in a simplified manner, and the antioxidant capacity of araçá, guabijú, and guabiroba fruits, which will be discussed separately.

### 5.1. Phenolic Compounds Present in Guabijú

In the case of guabijú (Table 1), even though there are few studies in the literature, these are expressive, since the phenolic profile of the fruit was detailed by Machado et al. [8] and Spinelli et al. [40].

According to Machado et al. [8], the free and bound phenolic compounds present in the three parts of the fruit: peel, pulp, and seeds, were studied separately. They quantified 18 phenolic compounds using analytical standards. Using triple quadrupole (QqQ) and quadrupole time-of-flight (QToF), they identified 81 compounds belonging to the following phenolic classes: 8 hydroxybenzoic acids; 9 hydroxycinnamic acids; 8 coumarins; 11 flavones; 7 flavanones; 5 isoflavones; 24 flavonols; 6 flavanols; 1 stilbene; 1 tannin; and 1 proanthocyanidin.

The authors observed a higher concentration of bound phenolic compounds, compared to free ones, mainly in the fruit’s skin and seeds. The pulp presented the lowest concentrations of these compounds, reinforcing the need for studies that evaluate all parts of the fruit, not just the edible ones.

Spinelli et al. [40] also investigated the phenolic compounds bound to the fruit, where they identified six phenolic classes, which they classified as approximately 30% phenolic acids, 24% hydrolyzable tannins, 18% flavanols, 9% flavonols, 8% anthocyanins, and 5% condensed tannins. The phenolic acids and hydrolyzable tannins were the major compounds in the fruit.

Seraglio et al. [42] studied the fruit pulp and seed using a triple quadrupole mass/charge analyzer, where similarities were also observed in the quantified phenolic compounds.

Some identified compounds were the same in both studies [7,40], such as gallic acid, ellagic acid, catechin, epicatechin, malvidin-3-glucoside, and delphinidin-3-glucoside, which contributed significantly to the fruit’s phenolic profile.

Gallic acid, commonly found in considerable concentrations in both fruits in this review, has bioactive and functional properties, including biological, antioxidant, antibacterial, and anti-inflammatory properties [47,48,49]. Ellagic acid, also a prominent compound in these native fruits, has antioxidant, antimutagenic, and anticancer properties. Studies have shown that it can inhibit the development of cancer cells and also mitigate metabolic complications related to obesity [50,51,52].

For guabijú, the major anthocyanin is malvidin-3-glucoside, followed by delphinidin-3-glucoside. Malvidin-3-glucoside is also the major anthocyanin found in blueberries (*Vaccinium* spp.). Blueberries belong to a different family (Ericaceae), but they have phenolic compounds similar to those of guabijú [53].

Anthocyanins are natural pigments and also promising candidates for the prevention of diseases related to oxidative stress, as well as having antimicrobial properties and biological and pharmacological potential [54,55,56].

Based on these studies, it can be stated that guabijú has a well-defined and broad phenolic profile, meeting the scientific needs regarding the fruit. However, further studies on it are still needed in other areas, such as bioaccessibility and bioavailability.

### 5.2. Phenolic Compounds Present in Guabiroba

For guabiroba (Table 1), Machado et al. [9] recently presented in detail the phenolic compounds present in the fruit, separately evaluating the peel, pulp, and seeds. This reference is the only study to date to perform this type of analysis for the fruit. During quantification using the triple quadrupole (QqQ) method, the authors observed that the seeds and pulp of the fruit have higher concentrations of bound phenolic compounds compared to the peel. Ellagic and gallic acids were the matrix-bound compounds that presented the highest proportions in the fruit. Regarding compound identification using the quadrupole time-of-flight (QToF) method, the authors identified 62 compounds: 7 phenolic acids, 44 flavonoids, 7 coumarins, 2 stilbenes, and 2 tannins.

Other studies on the fruit only carried out tests to estimate its antioxidant capacity, evaluating the pulp [43], seeds [44], peel and pulp [46], as well as the whole fruit [45].

It is observed in Table 1 that some fruits have similar phenolic compounds, such as guabijú and guabiroba, which both contain phenolic acids. When considering the flavonoid class, it is noted that guabijú and guabiroba also have similar compounds, such as catechin, epicatechin, epigallocatechin, and quercetin [8,9,11]. For guamirim (*Myrcia oblongata* DC.), the flavonoids catechin, epigallocatechin, and quercetin were also characterized [57].

Flavonoids found in fruits have anticancer, antioxidant, anti-inflammatory, and antiviral properties, as well as potential applications in cancer prevention, cardiovascular disease prevention, and neuroprotection [58,59,60].

Of the fruits discussed, guabiroba has the same number of published studies as guabijú; however, they are simpler studies, demonstrating the need for more research on it, to create a more complete phenolic profile of it, as well as studies that evaluate its bioaccessibility/bioavailability, among other areas of science.

### 5.3. Phenolic Compounds Present in Araçás

For araçás (Table 1), the literature contains relevant studies [7,36,37] providing more comprehensive information about the phenolic compounds present in the fruits.

According to Machado et al. [7], they evaluated the free and bound phenolic compounds in all parts of the fruit (peel, pulp, and seed) separately in two araçá morphotypes: red and yellow. The authors observed that cyanidin-3-glucoside is the main anthocyanin present in the peel and pulp of the red morphotype, followed by petunidin-3-galactoside. Furthermore, they also quantified 14 phenolic compounds, with matrix-bound compounds presenting the highest proportions in the fruits. The primary compound in the yellow morphotype was catechin, and in the red morphotype, gallic acid, indicating that even though the fruits are of the same species, they present differences in their phenolic profile.

In their research, Pereira et al. [36] investigated the peel and pulp of the two araçá morphotypes, where they identified 10 phenolic compounds, including vanillic, syringic, *p*-coumaric, 4-hydroxybenzoic acids, and flavonoids, such as catechin, kaempferol, myricetin, quercetin, and anthocyanins, such as cyanidin-3-glucoside and malvidin-3-glucoside.

Mallmann et al. [37] evaluated the free and bound phenolic compounds of the two araçá morphotypes using whole fruits. The main compounds identified were gallic, ellagic, ferulic, and *p*-coumaric acids, quercetin and its derivatives, as well as ellagitannins and hydrolyzable tannins, represented by castalagin and vescalagin. The major anthocyanin identified was cyanidin-3-hexoside, in red araçá.

Araçás have compounds similar to the ”Rio Grande cherries” (*Eugenia involucrata* DC) such as catechin, epicatechin, taxifolin, and *p*-coumaric acid [7,61]. Studies on uvaia (*Eugenia pyriformis* Cambess), another fruit native to the Myrtaceae family, have detected compounds similar to araçá, such as catechin, epicatechin [62], gallic and *p*-coumaric acids, and quercetin [63]. In the analysis of anthocyanins, both red araçá and jabuticaba present cyanidin-3-glucoside as the primary compound in the fruit peel [7,64].

Of the three fruits covered in this review, araçá has the most significant number of studies available in the literature, covering a complete phenolic profile of the fruit. However, there are still gaps regarding its bioavailability/bioaccessibility, as observed for the other fruits.

### 5.4. Phenolic Similarities Between the Fruits Discussed

Evaluating the three fruits covered in this review—guabijú, guabiroba, and araçá—it can be concluded that they share some similarities in their phenolic compounds, as can be seen in Table 1.

In both fruits, gallic and ellagic phenolic acids can be observed in all their parts, as well as in their free and bound forms. Regarding these compounds, it is noteworthy that the bound forms were found in higher concentrations when compared to the free forms.

Regarding flavonoids, catechin and its derivatives epicatechin and epigallocatechin, as well as quercetin, can be observed in both fruits, demonstrating that, despite being distinct fruits, they share certain similarities, in addition to their berry shape.

Another important fact is that the antioxidant capacity assessed by the various authors cited, in this case, differs between the fruits, partly because the tests used are also different in some cases. However, they all have considerable antioxidant capacity, thus demonstrating the importance of studies on native fruits and their possible uses for human health.

## 6. Conclusions

This review highlights the potential of native Brazilian fruits from the Myrtaceae family as abundant sources of phenolic compounds, with a focus on guabijú, guabiroba, and araçá. Despite the lack of more detailed research on the phenolic composition of these fruits, the available information suggests that they possess significant antioxidant capacity and potential applications in food, medicine, and cosmetics.

Advances in extraction and analysis techniques, such as the use of chemical hydrolysis to release bound compounds and the combination of liquid chromatography with mass spectrometry, have enabled a more accurate and detailed characterization of these metabolites present in fruits.

Expanding research into native fruits is essential for the sustainable use of these plant resources, contributing not only to the development of new products with functional and natural appeal but also to the enhancement of national biodiversity and the promotion of food and nutritional security in Brazil.

## Figures and Tables

**Table 1:** Studies available in the literature on the phenolic compounds and antioxidant capacity of Araçá, Guabijú, and Guabiroba fruits.

Fruits	Fraction of Fruit Studied	Test	Identification/Quantitation	Phenolic Compounds Found	Phenolic Compound Content/Antioxidant Capacity	References
Red and Yellow Araças	Pulp	Folin–Ciocalteu; DPPH; ABTS	-	-	49.83 mg GAE/100 g; 68.83 mg GAE/100 g; 958.51 mg AAE/100.	[34]
	Peel, Pulp, and Seed were evaluated separately	ORAC	LC-ESI- QqQ -MS/MS	Gallic Acid; Protocatechuic Acid; 4-Hydroxybenzoic Acid; Chlorogenic Acid; Catechin; Caffeic Acid; Vanillic Acid; Epicatechin; Syringic Acid; Acid *p*-Coumaric; Ferulic Acid; Ellagic Acid; Taxifolin; Quercetin-3-Glycoside; Pelargonidin-3-Glycoside; Cyanidin-3-Glycoside; Malvidin-3-Glycoside; Delphinidin-3-Glucoside; Peonidin-3-Glucoside.	-	[7]
	Whole Fruits	Folin–Ciocalteu; Total Flavonoids; FRAP; DPPH	HPLC-PDA	-	Hydroxybenzoate derivatives (21.45 mg GAE/50 mL); Hydroxycinnamate derivatives (22.84 mg CAE/50 mL); Flavanols (7.93 mg ME/50 mL); Anthocyanins (2.11 PEE/50 mL).	[35]
	Peel and Pulp	DPPH; Folin–Ciocalteu	HPLC-QToF-MS.	Catechin; Kaempferol; Myricetin; Cyanidin-3-0-glucoside; Malvidin-3-0-glucoside; Vanillic Acid; Ellagic Acid; Syringic Acid; *p*-coumaric Acid; 4-hydroxybenzoic Acid.	-	[36]
	Whole Fruits	ORAC	LC-QToF-MS/MS.	A total of 43 and 45 phenolic compounds were identified from yellow and red araça, respectively.	-	[37]
	Whole Fruits	DPPH; Folin–Ciocalteu	UV/Vis (Anthocyanins and Carotenoids).	-	603.1 mg CLEA/100 g; 29.3 CYE/100 g.	[38]
	Pulp	DPPH; Folin–Ciocalteu;	UV/Vis (Anthocyanins and Carotenoids); HPLC.	Epicatechin; Gallic Acid; *p*-coumaric Acid; Ferulic Acid; Myricetin; Quercetin.		[39]
Guabijú	Peel, Pulp, and Seed were evaluated separately	ORAC	LC-ESI-QToF-MS/MS; LC-ESI- QqQ -MS/MS	A total of 81 phenolic compounds were identified; Quantified: Catechin; Epicatechin; Epigallocatechin; Quercetin; Myricetin; Taxifolin; Quercetin-3-Glycoside; Protocatechuic Acid; 4-Hydroxybenzoic Acid; Vanillic Acid; Syringic Acid; Ellagic Acid; Gallic Acid; Chlorogenic Acid; Caffeic Acid; *p*-Coumaric Acid; Ferulic Acid; *trans*-cinnamic Acid; Delphinidin-3-Glycoside; Petunidin-3-Glycoside; Cyanidin-3-Glycoside; Pelargonidin-3-Glycoside; Peonidin-3-Glycoside; Malvidin-3-Glycoside.	-	[8]
	Peel and Pulp	-	HPLC-ESI-QToF-MS/MS.	67 phenolic compounds were identified	-	[40]
	Peel	DPPH; ABTS	UV/Vis (Total Anthocyanins and Total Flavonoids).	-	10.54 mg GAE/100 g; 248.96 mg CYE/100 g.	[41]
	Peel and Pulp	DPPH; Folin–Ciocalteu; FRAP	UV-Vis (Total Anthocyanins); LC-ESI-MS/MS	Catechin; Epigallocatechin; Epicatechin; Kaempferol; Naringenin; Quercetin; Caffeic Acid; Chlorogenic Acid; *p*-Coumaric Acid; Ferulic acid; Gallic acid; 4-Hydroxybenzoic acid.	-	[42]
	Whole fruit	DPPH; ABTS	HPLC (Anthocyanins).	-	51.7% Cyanidin-3-glucoside; 60% Malvidin-3-glucoside.	[15]
Guabiroba	Peel, Pulp, and Seed were evaluated separately	ORAC	LC-ESI-QToF-MS/MS; LC-ESI- QqQ -MS/MS.	62 phenolic compounds were identified; Quantified: Catechin; Epicatechin; Epigallocatechin; Quercetin; Quercetrin; Quercetin-3-Glycoside; Myricetin; Kaempferol-3-Rutinoside; Ellagic Acid; Gallic Acid; Protocatechuic Acid; 4-Hydroxybenzoic Acid; Vanillic Acid; Caffeic Acid; Ferulic Acid.	-	[9]
	Pulp	DPPH	-	-	Antioxidant activity assessed by free radical scavenging (DPPH).	[43]
	Seeds	Folin–Ciocalteu; Total Flavonoids; DPPH; FRAP	-	-	68.58 mg GAE/100 g; 8.10 mg QEE/100 g.	[44]
	Whole Fruit	Folin–Ciocalteu; DPPH	-	-	Antioxidant activity assessed by free radical scavenging (DPPH); 39.12 mg GAE/100 g.	[45]
	Peel and Pulp	DPPH; ABTS	-	-	1353.7 mg of DPPH/g; 505.9 μmol/L Trolox Equivalents/g.	[46]

GAE: gallic acid equivalents; AAE: ascorbic acid equivalents; CAE: caffeic acid equivalents; ME: Myricetin equivalents; PEE: pelargonidin-3-glucoside equivalents; CLEA: chlorogenic acid equivalents; CYE: cyanidin-3-glucoside equivalents; QEE: quercetin equivalents.
